# Long-term exposure of bacterial and protozoan communities to TiO_2_ nanoparticles in an aerobic-sequencing batch reactor

**DOI:** 10.1088/1468-6996/16/3/034609

**Published:** 2015-06-18

**Authors:** Chitpisud Supha, Yuphada Boonto, Manee Jindakaraked, Jirapat Ananpattarachai, Puangrat Kajitvichyanukul

**Affiliations:** Center of Excellence on Environmental Research and Innovation, Faculty of Engineering, Naresuan University, Phitsanulok, 65000, Thailand

**Keywords:** TiO_2_, antibacterial effects, wastewater, nanoparticles, microbial, inhibition

## Abstract

Titanium dioxide (TiO_2_) nanopowders at different concentrations (0–50 mg L^−1^) were injected into an aerobic-sequencing batch reactor (SBR) to investigate the effects of long-term exposure to nanoparticles on bacterial and protozoan communities. The detection of nanoparticles in the bioflocs was analyzed by scanning electron microscopy, transmission electron microscopy, and energy-dispersive x-ray spectroscopy. The SBR wastewater experiments were conducted under the influence of ultraviolet light with photocatalytic TiO_2_. The intrusion of TiO_2_ nanoparticles was found both on the surface and inside of the bioflocs. The change of microbial population in terms of mixed liquor-suspended solids and the sludge volume index was monitored. The TiO_2_ nanoparticles tentatively exerted an adverse effect on the microbial population, causing the reduction of microorganisms (both bacteria and protozoa) in the SBR. The respiration inhibition rate of the bacteria was increased, and the viability of the microbial population was reduced at the high concentration (50 mg L^−1^) of TiO_2_. The decreasing number of protozoa in the presence of TiO_2_ nanoparticles during 20 days of treatment with 0.5 and 1.0 mg L^−1^ TiO_2_ is clearly demonstrated. The measured chemical oxygen demand (COD) in the effluent tends to increase with a long-term operation. The increase of COD in the system suggests a decrease in the efficiency of the wastewater treatment plant. However, the SBR can effectively remove the TiO_2_ nanoparticles (up to 50 mg L^−1^) from the effluent.

## 1. Introduction

Nanotechnology is becoming an attractive discipline that draws many scientists, researchers, and engineers to explore, investigate, and create many innovations. Nanoparticles include metal oxide nanopowders that are mainly titanium dioxide (TiO_2_), zinc oxide (ZnO), silica (SiO_2_), alumina (Al_2_O_3_), and iron oxides (Fe_3_O_4_, Fe_2_O_3_). These nanomaterials have some superior physicochemical properties than the bulk materials due to their nanoscale size. This small size is critical for the enhanced physical phenomena leading to different properties in chemical and biological reactions. As the advanced exploration of nanotechnology continues, a great number of consumer products containing nanoparticles have reached the markets. The nanoparticles from sunscreen, toothpaste, detergents, and other products are finally entering sewage systems. These nanoparticles exhibit antibacterial properties [1–3], disrupt microbial activities in activated sludge, and affect the efficiency of wastewater treatment plants [4, 5]. Among the many nanoparticles, titanium dioxide (TiO_2_) is one of the most widely used nanomaterials and is present in many personal products. The TiO_2_ nanoparticles may exert a negative impact on aquatic ecosystems, which are related to human health [6, 7]. When the TiO_2_ nanoparticles enter wastewater treatment plants, many adverse effects may occur. The physicochemical stability and biological stability of the activated sludge bioflocs are possibly threatened by exposure to these nanoparticles. TiO_2_ nanoparticles have been reported to show toxicity towards bacteria [8] under ultraviolet (UV) irradiation [9, 10] due to the generation of reactive oxygen species (ROS) [11, 12]. These ROS can disrupt cellular membranes, causing damage to bacterial cells [10, 13]. In addition, the nanoscale size may contribute to the toxicity of TiO_2_ nanoparticles, as well as the inhibitory effects of TiO_2_ nanoparticles that can be observed in the absence of light [8].

The likely concentration of TiO_2_ nanoparticles in the effluent of wastewater treatment is reported to be around 0.01–0.2 mg L^−1^ [4, 14]. Kiser *et al* also showed that 96% of the TiO_2_ was possibly removed from the wastewater treatment plant when the TiO_2_ concentration was below 2 mg L^−1^ [4]. Therefore, the TiO_2_ concentration in wastewater would be around 0.1–2.0 mg L^−1^. Although many previous works have reported the effect of TiO_2_ nanoparticles on the bacteria, more research studies are needed to better understand the impact of these nanoparticles on other microbial populations and the stability of bioflocs in the system. In addition, the changes in the microbial population in the system and the stability of bioflocs that tentatively exert an effect on the treatability performance of wastewater treatment plants should be investigated.

In the present investigation, the effect of TiO_2_ nanoparticles on biological wastewater treatment was examined in activated sludge. The low concentrations of TiO_2_ at 0.05 and 1.0 mg L^−1^ were selected to represent the likely concentration of the real wastewater to be treated. The concentrations of TiO_2_ at 10, 30, and 50 mg L^−1^ were also investigated to demonstrate the impact of high loading rates of nanoparticles on water treatment plants. The changes in the microbial community, the biofloc, and the treatability performance of the wastewater treatment plant were the principal investigated topics. The COD of the effluent was measured to indicate the treatability performance of the investigated aerobic-sequencing batch reactor (SBR). This parameter is the indirect measurement of the amount of pollution that cannot be oxidized biologically in the wastewater. All results were incorporated to demonstrate the impact of TiO_2_ nanoparticles on the microbial community, as well as its effect on the effluent that indicated the performance of the wastewater treatment plant.

## 2. Materials and methods

### 2.1. Materials

Titanium tetraisopropoxide (TTiP), C_6_H_12_O_6_, NH_4_HCO_3_ and KH_2_PO_4_ were purchased from Aldrich Chemicals. Ethanol (EtOH), HNO_3_, H_2_SO_4_, NaOH, MgSO_4_•7H_2_O, CaCl_2_•2H_2_O, MnCl_2_•4H_2_O, ZnSO_4_•7H_2_O, CuCl_2_•2H_2_O, Na_2_MoO_4_•2H_2_O, FeSO_4_•7H_2_O, and KI were obtained from Merck Chemicals. All of the reagents were of analytical grade. Ultrapure water from Millipore, Billerica, was used for the wastewater experiment, and 18 M*Ω* deionized water (H_2_O) was used to prepare the solutions.

### 2.2. TiO_2_ synthesis and suspension

A modified sol–gel method was used to synthesize TiO_2_ with a molar ratio of 1:20:1:1 for TTiP:EtOH:HNO_3_:H_2_O as described by Ananpattarachai *et al* [15]. First, TTiP was dissolved in EtOH, and the solution was stirred for 30 min. In the second solution, EtOH was mixed with H_2_O that contained HNO_3_. Precipitation readily occurred after both portions were mixed. The homogeneous transparent solution was then kept for 30 min at 4 °C with stirring before undergoing the drying process. After drying at 100 °C for 90 min, the powder was collected and calcined at 500 °C in an electric furnace. The TiO_2_ nanoparticles were collected after they were cooled to room temperature. The TiO_2_ nanoparticles obtained from this procedure were previously reported for photocatalytic degradation of 2-chlorophenol [15].

The particle size distributions and the hydrodynamic radii of the nano-TiO_2_ particles were determined by dynamic light scattering (DLS) at a 90-degree angle by using a Zetasizer Nano series instrument (Malvern, UK). The concentrations of Ti in the bioflocs and effluent were measured by inductively coupled plasma optical emission spectroscopy (ICP-OES) [16]. The TiO_2_ extraction method has been described previously [17]. For the wastewater experiment, a stock solution of 1.0 g L^−1^ TiO_2_ was prepared by dispersing 0.1 g of TiO_2_ nanoparticles in ultrapure water with sonication for 30 min (50 W L^−1^ at 40 kHz). The test solutions of TiO_2_ nanoparticles were prepared immediately prior to use by diluting this stock solution with synthetic wastewater.

### 2.3. Activated sludge wastewater treatment plant

The synthetic wastewater contained 400.0 mg L^−1^ of C_6_H_12_O_6_, 40.0 mg L^−1^ of NH_4_HCO_3_, and 8.0 mg L^−1^ of KH_2_PO_4_. It also contained 70.0 mg L^−1^ MgSO_4_•7H_2_O, 20.0 mg L^−1^ CaCl_2_•2H_2_O, 17.5 mg L^−1^ FeSO_4_•7H_2_O, 0.15 mg L^−1^ MnCl_2_•4H_2_O, 0.15 mg L^−1^ ZnSO_4_•7H_2_O, 0.07 mg L^−1^ CuCl_2_•2H_2_O, 0.03 mg L^−1^ Na_2_MoO_4_•2H_2_O, and 0.03 mg L^−1^ KI. The experiments were conducted in six lab-scale aerobic SBRs (five parallel samples were spiked with different concentrations of TiO_2_ nanoparticles, and one reactor was used as the control). A 10-watt UV lamp (TUV T8, Phillips) was used as the light source for each reactor. The SBR reactors were made of glass with a working volume of 10 L. The UV lamp was placed on top of the reactor without any baffle between the lamp and the wastewater. Activated sludge from a domestic wastewater treatment plant (Phitsanulok, Thailand) was used as the inoculum. The SBRs were operated with a cycle time of 24.0 h at room temperature. Before being doped with TiO_2_ nanoparticles, the six SBRs were in stable operation for one month and were monitored for the related parameters. The TiO_2_-containing reactors were run for another month to achieve a quasi-steady state. The SBR was operated with a food-to-mass ratio of 0.25. The mixed liquor- suspended solids (MLSS), sludge volume index (SVI), influent COD, and effluent COD were measured in every cycle following the standard methods [18]. The residual Ti in bioflocs and effluent was also analyzed routinely by atomic absorption spectroscopy (Varian, Model AA220Z) as described in the standard methods [18].

### 2.4. Wastewater and sludge characteristics

Wastewater and sludge characteristics were analyzed in duplicates following the established standard methods (APHA 1999). For biofloc morphology analysis by scanning electron microscopy (SEM) and energy-dispersive x-ray spectroscopy (EDS), bioflocs were sampled from the SBR. They were washed with phosphate buffer solution (PBS) and soaked overnight in 2.5 wt% glutaraldehyde. The treated sample was washed again with PBS and dehydrated with an ethanol gradient (25, 50, 75, 90 and 99%) [18]. The samples were resuspended in 99% ethanol before dropped onto the SEM specimen mount holder and dried in a desiccator. The dried samples were coated with platinum for SEM analysis using a JOEL JSM-5310 microscope operated at 15 kV.

For transmission electron microscopy (TEM) analysis, samples were obtained from the sludge with 1.0 mg L^−1^ of TiO_2_ concentration. The sample was fixed in 2% glutaraldehyde and 0.05 M sodium cacodylate buffer (pH 7.2) for 2 h and washed three times with 0.05 M sodium cacodylate buffer. The samples were dried in a desiccator before analysis using a Hitachi H-8100 TEM operated at 200 kV.

### 2.5. Microbial community analysis

The protozoan community was monitored as described previously [19]. A 1.0 mL aliquot of sample was injected into a culture tube (16 by 150 mm). Two drops of brilliant green dye (2 g of brilliant green dye and 2 mL of glacial acetic acid diluted to 100 mL with distilled water) were added, and the contents were mixed and allowed to stand for at least 4 h. Then, 9 mL of 30% glycerol solution was added, resulting in a 1:20 dilution of the original protozoa contents. The diluted sample was pipetted into a Sedgewick–Rafter counting chamber. Protozoa in the samples were counted at a magnification of ×100 with a counting grid 0.5 mm^2^. By using a calibrated microscope stage, 50 grids, evenly spaced over the entire chamber surface, were counted. The chamber was then rotated by 180 degrees, a second 50-grid count was made, and these two counts were averaged [19].

The bacterial enumeration was performed by the drop plate method [20] for heterogeneous plate count. The samples (100 *μ*L) were taken at time intervals during wastewater treatment. A series of 10-fold dilutions of the samples were performed. Ten *μ*L of each dilution was plated on R2A agar (Voigt Global Distribution, Inc., Kansas, USA) in triplicate. Plates were incubated at 31 °C for 24 h and held at room temperature for another seven days. Counting was performed after seven days for the total number of bacteria. The lower detection limit was 10^2^ CFU/mL (CFU = colony-forming unit). All assays were expressed as mean ± standard deviation. An analysis of variance (ANOVA) was used to test the significance of the results, and *p* < 0.05 was considered to be statistically significant.

The bacterial activity was assessed by the oxygen uptake rate (OUR) measurements. Aliquots (60 mL) of the mixed liquor were taken from the aeration zone for the OUR measurement by extant respirometry, following the procedure described previously [21]. The OUR was also used to estimate the inhibition rate using the following equation:

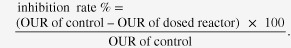


Polymerase chain reaction denaturing gradient gel electrophoresis (PCR-DGGE) fingerprinting was performed to examine the bacterial communities. For PCR-DGGE analysis, 10 mL of mixed liquor was centrifuged for 5 min at 1000 g, and the supernatant was decanted, frozen and stored at −20 °C. The DNA extraction was performed with a QIAamp DNA Mini Kit (Qiagen, Valencia, California, USA) to obtain the fingerprint patterns and the band similarities. To minimize variations in DNA extraction, templates used for PCR amplification were prepared by mixing the DNA extracted in triplicate for each sample. The resultant image was analyzed using the Quantity One 4.6.2 program (Bio-Rad, USA).

## 3. Results and discussion

### 3.1. Characterization of TiO_2_ nanoparticles and ROS generation

**Figure 1.**
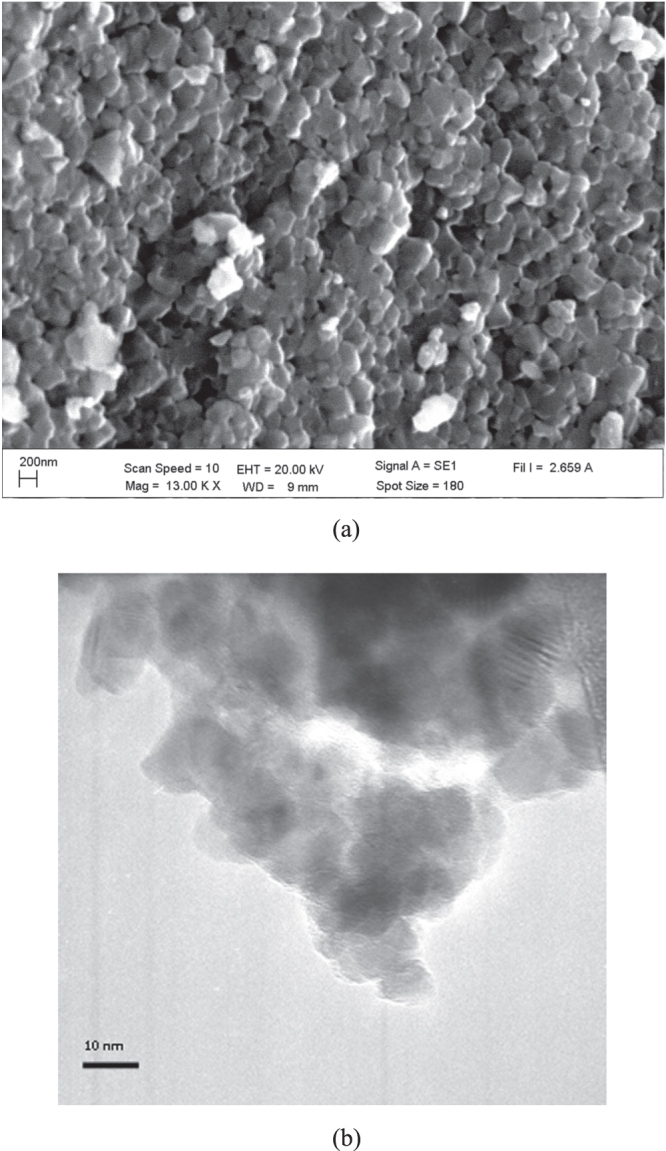
(a) SEM and (b) TEM images of TiO_2_ nanoparticles.

Figures 1(a) and (b) show SEM and TEM images of TiO_2_ nanoparticles. From TEM analysis, TiO_2_ nanoparticles (with 97% purity) have a crystal size of 10–20 nm and consist of 66% anatase and 34% rutile phases. The average hydrodynamic diameter of the particles from DLS measurement (in ultrapure water medium) increased with the concentration of TiO_2_ nanoparticles; it was 85 ± 9 nm, 125 ± 18 nm, 525 ± 21 nm, 755 ± 28 nm and 1038 ± 23 nm for TiO_2_ concentrations of 0.05 mg L^−1^, 1 mg L^−1^, 10 mg L^−1^, 30 mg L^−1^ and 50 mg L^−1^, respectively.

**Figure 2.**
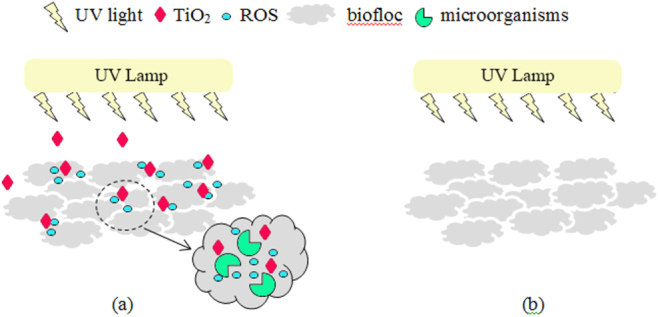
Schematic representation of TiO_2_-bioflocs interaction in an aerobic-sequencing batch reactor, (a) with TiO_2_ and UV light, the photocatalytic TiO_2_ nanoparticles dispersed in the bioflocs and ROS generated from UV illumination of the nanoparticles diffuse into the bioflocs and come into contact with the microorganisms; and (b) without TiO_2_, there is no exposure of TiO_2_ and ROS on the microorganisms.

In this present work, the TiO_2_ nanoparticles were loaded to the wastewater, and the treatment plant was irradiated by the UV light to study the impact of long-term exposure of irradiated TiO_2_ nanoparticles on bacterial and protozoan communities. These investigated nanoparticles have been proved to possess photocatalytic properties capable of degradation of 2-chlorophenol with 80 and 14% removal efficiency under UV and visible light, respectively [15]. The impact of UV light without TiO_2_ nanoparticles on the microorganisms in the wastewater treatment plant was also conducted as the control experiment. As reported previously in several studies [11, 12, 22], light absorption by the photocatalytic TiO_2_ can generate ROS, including hydroxyl radicals (OH^•^), superoxide radicals (O_2_^•−^), and hydrogen peroxide (H_2_O_2_). These radicals are considered to be the active components promoting the bactericidal effect [11, 12]. Their ability to disrupt cellular membranes and damage bacterial cells has been reported [10, 13]. Thus, the schematic scenarios for the treatment plants with and without TiO_2_ (or control experiment) are shown in figure 2. The impacts of the TiO_2_ nanoparticles under the UV light on the bioflocs and change of microorganisms are discussed in the following section.

### 3.2. Sorption of TiO_2_ by bioflocs and change of mixed liquor-suspended solids in the SBR

**Figure 3.**
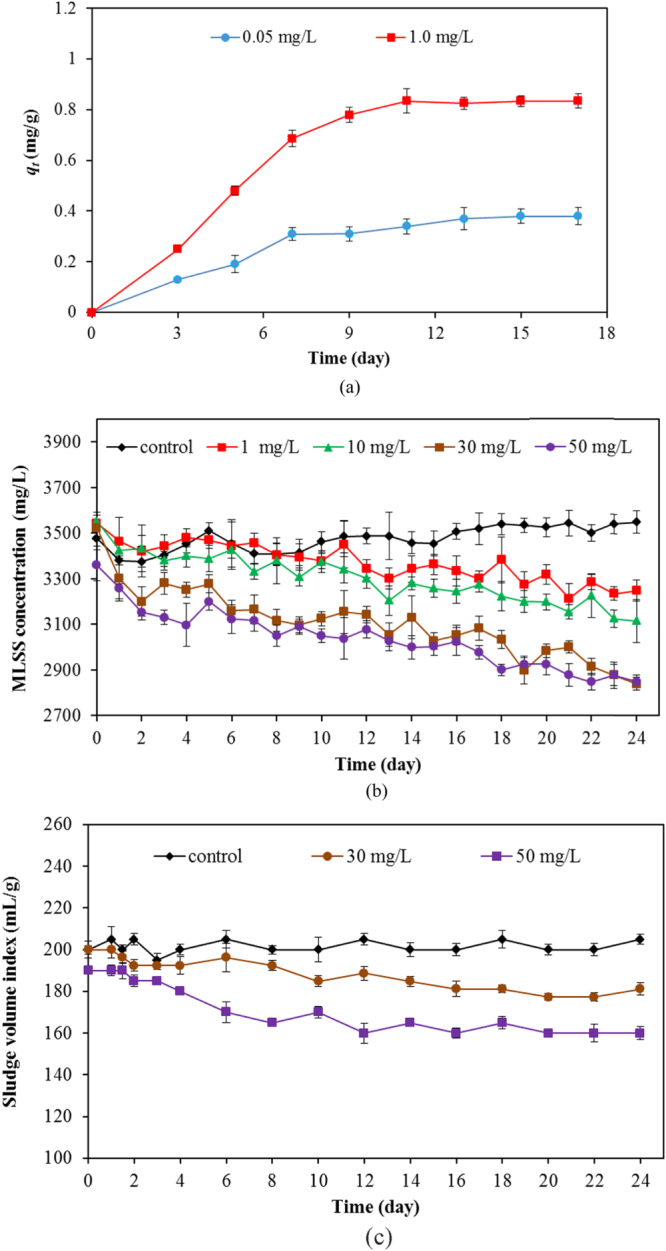
(a) time profile of Ti sorption on bioflocs of activated sludge, (b) change of MLSS concentration, and (c) change of SVI with time for various TiO_2_ concentrations.

The time profile of Ti adsorption on bioflocs of activated sludge is shown in figure 3(a). The term *q*_*t*_ represents the maximum sorption capacity of Ti on bioflocs in the unit of mg of Ti/g of MLSS. At a lower initial TiO_2_ concentration at 0.05 mg L^−1^, the adsorption of Ti on the activated sludge (*q*_*t*_) reached equilibrium sooner than the higher initial TiO_2_ concentration at 1.0 mg L^−1^. This behavior may due to the relative abundance of sorption sites available. The maximum sorption capacity was 0.382 and 0.834 mg g^−1^ for 0.05 and 1.0 mg L^−1^ TiO_2_ concentration, respectively. The sorption of TiO_2_ by bioflocs may be the major mechanism that causes the agglomeration of TiO_2_ nanoparticles in the bioflocs and results in the decrease in microorganisms in the treatment plant. To elucidate the impact of nanoparticles on the quantity of microorganisms in the system, the MLSS were assessed. This testing measures the concentration of suspended solids during the aeration stage of the activated sludge process. It consists mostly of microorganisms and non-biodegradable suspended matter. The SVI, which is the tendency indicator of activated sludge solids to become concentrated during the sedimentation or thickening process, was also determined. Change of MLSS (or SVI) is an important parameter indicating the performance of the wastewater treatment plant.

The MLSS changes that occurred in the investigated SBR with and without TiO_2_ are shown in figure 3(b). It was found that, without TiO_2_, MLSS was stable at 3400–3500 mg L^−1^. However, with the TiO_2_ addition, the MLSS decreased sharply. The concentrations of MLSS with 1, 10, 30, and 50 mg L^−1^ for 12 h in SBR were 3143, 3010, 2945, and 2796 mg L^−1^ on average. Figure 3(c) shows the change of SVI with time for SBR with and without TiO_2_. The SVI for the SBR without TiO_2_ nanoparticles was steady at 200 mL g^−1^. With TiO_2_ nanoparticles, the average SVI values were 165 and 170 mL g^−1^ for 30 and 50 mL g^−1^ of TiO_2_ concentration, respectively, during 12 days of activated sludge activity in the SBR system. Results from changes of MLSS and SVI shows that TiO_2_ nanoparticles tentatively exerted an adverse effect on the microbial population, causing the reduction of MLSS in the system. In addition, the decrease of the SVI value indicates the higher agglomerate of sludge or bioflocs in the system. Both changes of MLSS and SVI were possibly related to the change in the microbial community in the SBR system.

### 3.3. SEM and TEM of bioflocs with and without TiO_2_ nanoparticles

**Figure 4.**
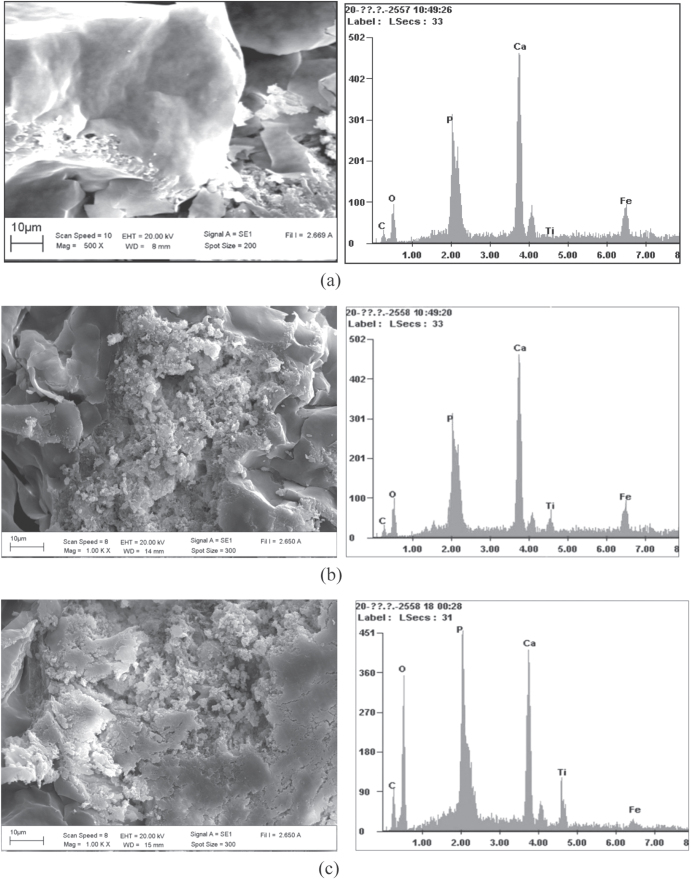
SEM images (left) and EDS spectra of bioflocs (right, *x*-scale is in keV). (a) without TiO_2_, (b) with 30 mg L^−1^ of TiO_2_ and (c) with 50 mg L^−1^ of TiO_2_.

**Figure 5.**
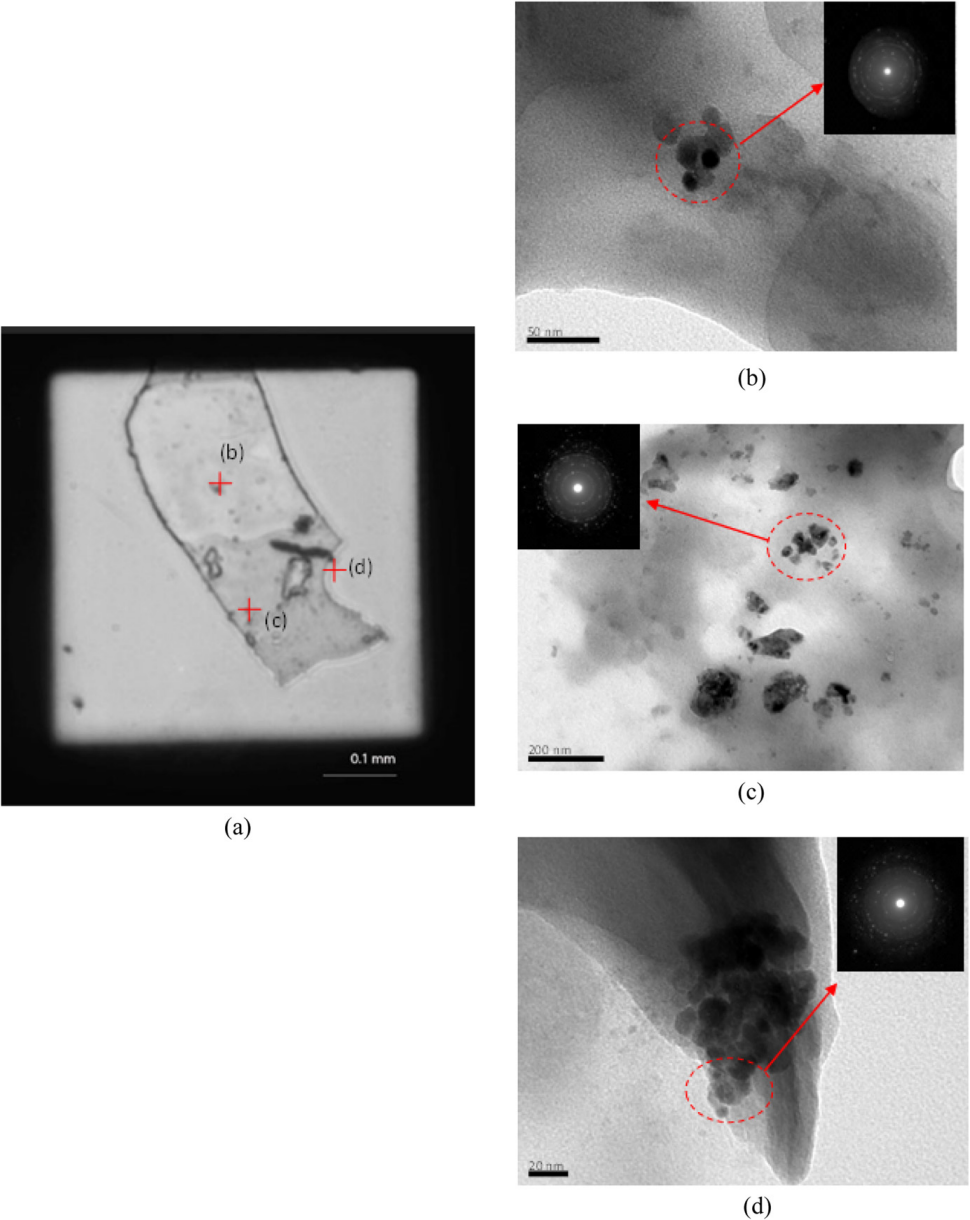
Optical microscopy image of protozoa on the TEM copper grid (a) and TEM images with electron diffraction patterns of TiO_2_ nanoparticles from different locations inside cell (b)–(d).

Analytical SEM was employed for imaging the detail of the surface of the sludge or bioflocs with and without the TiO_2_ nanoparticles as shown in figures 4(a)–(c). The surface morphology of the bioflocs without TiO_2_ nanoparticles was relatively smooth compared to other bioflocs in the presence of TiO_2_. Morphology of bioflocs with TiO_2_ revealed that the TiO_2_ nanoparticles were found both on the surface and inside the bioflocs. Energy-dispersive spectroscopy (EDS) data reveal the presence of Ti in the bioflocs. Apparently, with the increasing concentration of TiO_2_ in the SBR system, the Ti contents in bioflocs were also increased. The SEM images and EDS data clearly show that the activated sludge bioflocs were surrounded by the TiO_2_ nanoparticles. The TEM analysis with electron diffraction was also performed to detect the nanoparticles in the bioflocs with 1.0 mg L^−1^ TiO_2_ concentration as shown in figure 5. Figure 5(a) shows the optical microscopy image of the dead protozoa on the TEM copper grid. The nanoparticles were detected in different locations inside the cell by TEM analysis (b–d). The electron diffraction patterns of TiO_2_ from the red circle area in TEM analysis also demonstrated the existence of agglomerates of small nanoparticles in the cell. One previous study [23] reported that a high concentration of TiO_2_ can cause greater interruption to the cell than with low concentration. Our data demonstrated that the TiO_2_ nanoparticles interact with wastewater bioflocs and may be taken up by the protozoa. This result is possibly due to the bioflocs having accumulated much of the TiO_2_ particles presented in the influent over a long period by the activated sludge flocs.

### 3.4. Effect of TiO_2_ on changes in the bacterial community

**Figure 6.**
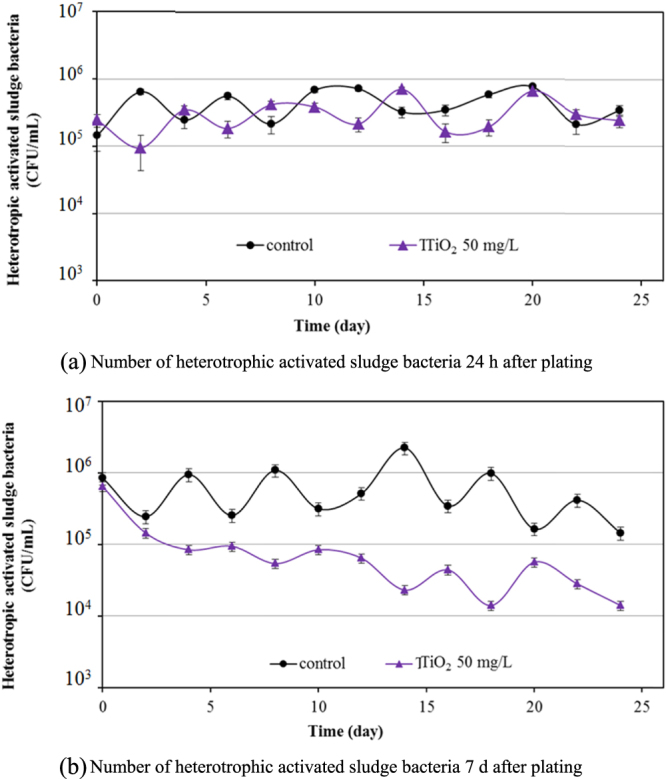
Effect of 50 mg L^−1^ TiO_2_ on heterotrophic activated sludge bacteria in activated sludge samples.

The impact of TiO_2_ on the bacterial community was studied by the culturability of heterotrophic bacteria in activated sludge. The heterotrophic plate count after 24 h (figure 6(a)) indicated the fast-growing heterotrophic bacteria in activated sludge samples, and that after 7 days plating (figure 6(b)) indicated the total number of viable bacteria in the samples. Initially, the fast-growing and total bacteria in sludge samples were around 4.44 × 10^5^ and 8.45 × 10^5^ CFU mL^−1^, respectively. During the 25 days of wastewater treatment in the control experiment without TiO_2_ nanoparticles, the number of the fast-growing heterotrophic bacteria was stabilized at around 3.4 × 10^5^ CFU mL^−1^. The number of bacteria in sludge samples with 50 mg L^−1^ TiO_2_ nanoparticles was similar during 25 days of treatment, revealing that the nanoparticles had no significant impact on the heterotrophic cell culturability of the sludge samples. However, the effect of TiO_2_ nanoparticles on the total number of viable bacteria after seven days plating was clearly seen (figure 5(b)) with significant statistical differences. The 50 mg L^−1^ TiO_2_ caused a loss of about 1.5log units in the total number of heterotrophic bacteria. The heterotrophic plate count started to decrease from around two days of the wastewater treatment. It was reported that this contact time may be associated with the time taken for nanoparticles to diffuse into the activated sludge flocs. Tiede *et al* [24] and Sun *et al* [25] reported that 6–8 h contact time was needed for Ag nanoparticles to partition into the sewage sludge. In this TiO_2_ study, two days contact time was required for the partitioning of TiO_2_ nanoparticles into the sludge.

A PCR-DGGE analysis was also used in this work to obtain the information on the bacterial community. Although PCR-DGGE is a culture-independent molecular finger printing technique to observe microbial diversity and community shifts, it is limited in detecting base sequence differences that are less than 95% [26]. In our work, the comparison of community structure of bacteria with and without TiO_2_ nanoparticles shows that there is no significant difference in the microbial community. The addition of TiO_2_ nanoparticles (at 0.05 mg L^−1^ and 1.0 mg L^−1^) did not cause a substantial change in the community. Many previous works have discussed the toxicity of TiO_2_ nanoparticles to bacteria due to the generation of ROS and the disruption of the cellular membrane, resulting in cell damage [9, 10, 13]. TiO_2_ nanoparticles can be internalized by bacterial cells and exert oxidative stress that damages the DNA and results in cell death [2]. Zheng *et al* [27] also showed that 50 mg L^−1^ TiO_2_ nanoparticles cause a decrease in the biodiversity of the sludge microbial community. However, in our work, we cannot detect this effect of TiO_2_ nanoparticles on the bacterial community. It is possible that the microbial community shifts in the unsettled sludge samples were not detected by DGGE in our work. It was discussed previously that DGGE was not sensitive enough to distinguish all sequence changes in 16S rRNA gene fragments [26, 28].

Our work demonstrated that the nanoparticles diffused and associated into the bioflocs. The association of nanoparticles with biomass is likely to occur in one or two steps. In the first step, the nanoparticles are adsorbed to bacterial surfaces. Some previous works suggested that the phenomenon is driven by electrostatic attraction [29–31]. However, up to the present time, the specific mechanism(s) responsible for the adsorption of nanoparticles to bacterial surfaces is still unknown. After the adsorption of nanoparticles to the cell surface, a possible second step is the uptake of nanoparticles into the cell. Many mechanisms such as passive diffusion or facilitated transport across an intact membrane or diffusion across a disrupted membrane may play a role in this step [32]. These steps lead to the toxicity of the nanoparticles on the microbial community in the wastewater. The impact of the TiO_2_ nanoparticles on bacterial activity can be reported as a percentage of inhibition rate calculated from the OUR as shown in table 1. The average control respiration rates were 6.8 ± 0.6 mg O_2_ L^−1^h^−1^ for the control experiment without TiO_2_ nanoparticles. In the presence of TiO_2_ nanoparticles, the respiration rates progressively decreased when the TiO_2_ concentration increased, e.g., at 1.0 g L^−1^ of TiO_2_, the respiratory activity was 4.0 ± 0.5 with 41.2 ± 0.7% inhibition rate. The lowest respiration rate was observed when the TiO_2_ concentration was the highest, demonstrating that TiO_2_ nanoparticles imposed toxicity in the form of respiration inhibition on the microbial population.

**Table 1.** Respiration rate (OUR) and inhibition rate of bioflocs in activated sludge (using ANOVA and statistical significant at *p* < 0.05).

Concentration of TiO_2_ (mg L^−1^)	Respiration rate (mg O_2_ L^−1^h^−1^)	Inhibition rate (%)	*p* value
Control	6.8 ± 0.6	0.0 ± 0.5	0.512
0.05	5.6 ± 0.2	18.5 ± 0.7	0.534
1	4.0 ± 0.5	41.2 ± 0.7	0.510
10	3.3 ± 0.5	51.9 ± 0.4	0.632
30	2.7 ± 0.4	60.3 ± 0.2	0.587
50	2.0 ± 0.2	70.5 ± 0.3	0.531

### 3.5. Effect of TiO_2_ on changes in the protozoa community

**Figure 7.**
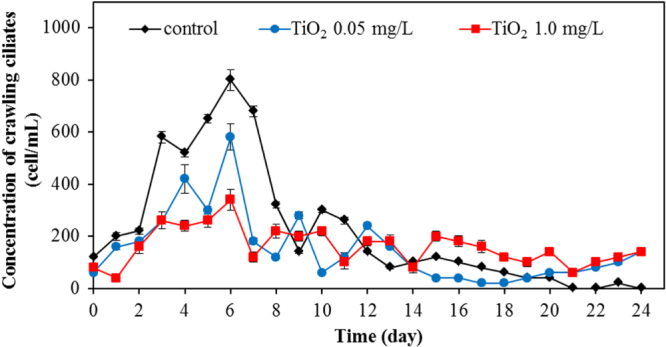
Changes of concentration of crawling ciliates for various TiO_2_ concentrations.

**Figure 8.**
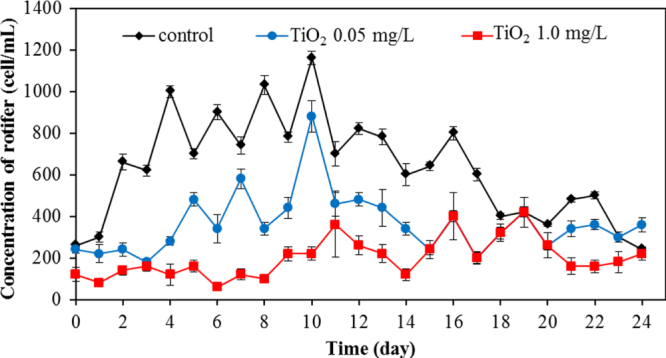
Changes of concentration of rotifer for various TiO_2_ concentrations.

**Figure 9.**
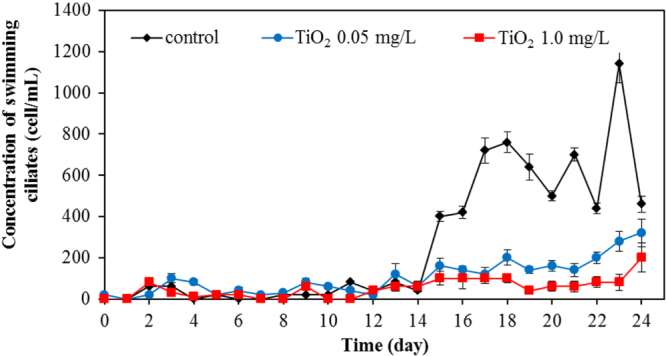
Changes of concentration of free-swimming ciliates for various TiO_2_ concentrations.

**Figure 10.**
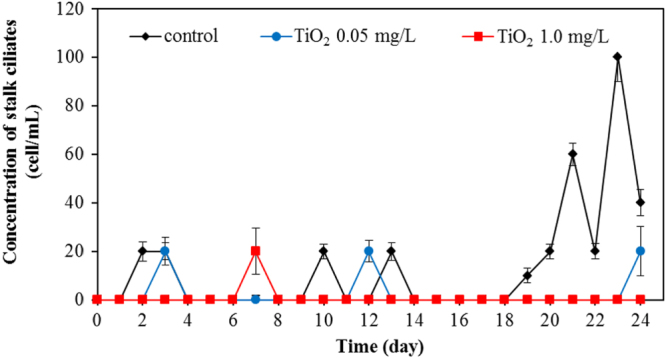
Changes of concentration of stalk ciliates for various TiO_2_ concentrations.

Apart from bacteria, protozoa are the next most important group of microbes in the wastewater. In this work, we also investigated the protozoa community. Protozoa also play a significant role in the efficient functioning of wastewater treatment plants. They are efficient at gathering microbes as food, and this improves the wastewater treatment performance, resulting in a lower organic load in the effluent of the treated wastewater. Currently, whereas many studies examined the bacterial community, relatively few have monitored the protozoan community. In this work, changes of major protozoa species found in the wastewater treatment plant were monitored and counted according to the previous study [18]. Changes in crawling ciliates, rotifer, free-swimming ciliates, and stalk ciliates are shown in figures 7–10, respectively. The statistical differences in the concentration of each protozoan with the application of the different amount of TiO_2_ concentration were observed.

During the earlier stage of our wastewater treatment, the dominant protozoa species were crawling ciliates (figure 7) and rotifers (figure 8). The crawling ciliates increased during the first five days before then decreasing with time. The pattern of changes in the population of these species during the wastewater treatment was similar both with and without TiO_2_ nanoparticles. However, it is obvious that the number of crawling ciliates was reduced with increasing TiO_2_ concentration.

For rotifers, this group was frequently found for the whole period of treating wastewater. Without TiO_2_ in the system, this species was detected in the range of 600–1200 cell/mL. However, in the presence of 0.5 mg L^−1^ of TiO_2_, the rotifer count was in the range of 200–900 cell mL^−1^. The rotifer count was at the relatively low level in the range of 30–400 cell/mL with 1.0 mg L^−1^ of TiO_2_. The rotifer numbers reduced sharply with a high concentration of TiO_2_ in the wastewater treatment plant. TiO_2_ nanoparticles also exerted an adverse effect on rotifer numbers as seen in figure 8. In the presence of 0.05 and 1.0 mg L^−1^ TiO_2_, the number of rotifers was drastically reduced, with the residual rotifer count less than 50% of that in the control (without TiO_2_) wastewater treatment plant. This adverse effect on the protozoan community is also shown in the monitoring of stalk ciliates (figure 10). With the 0.05 and 1.0 mg L^−1^ TiO_2_ in the wastewater, the stalk ciliates almost disappeared from the system.

In overall results, it can be clearly seen that TiO_2_ nanoparticles had an adverse impact on the microbial community. Numbers of all the investigated species of protozoa were reduced in the presence of TiO_2_. The higher the concentration of TiO_2_, the more adverse effect on the protozoan community was evident. Presumably, the TiO_2_ nanoparticles were readily ingested by the protozoa, as some previous work showed that there were a number of small particles inside the protozoa body [33]. The ciliated protozoa (crawling, free-swimming, and stalking ciliates) can ingest the nanoparticles via a cytostome. After capture, the nanoparticles may be contained in a spherical bubble or phagosome (food vacuole). It was reported that during the maturation steps of the phagosome, the hydrolytic enzymes participate in the phagolysosomal degradation of ingested particles [34]. The enzymes break down food into suitable units for metabolism. As the pH in the lumen of food vacuoles becomes acidic after vacuole formation [35], a metal oxide particle can undergo solution under these conditions. Mortimer *et al* [33] reported that *T*. *thermophila*, one type of ciliate, is capable of functioning after ingesting a large quantity of CuO nanoparticles. For rotifers, the disc cilia can create water currents to move food and particles into the mouth. The nanoparticles may pass down the esophagus into the mastax and the large brownish stomach where food is digested, and the indigestible remains pass into the short intestine [36]. The toxicity of TiO_2_ nanoparticles on these protozoa may occur from the generation of ROS in the same manner as with bacterial toxicity. The decreasing number of protozoa in the presence of TiO_2_ nanoparticles is clearly demonstrated in our work.

### 3.6. Treatability performance of SBR from COD and TiO_2_ removal

**Figure 11.**
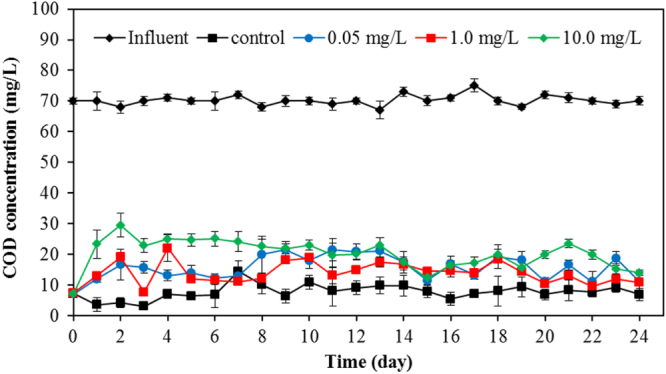
COD removal percentage of SBR variation of TiO_2_ concentrations in the effluent.

The COD removal efficiency was determined from the effluent of the SBR with and without TiO_2_ as shown in figure 11. As the TiO_2_ nanoparticles exerted an adverse effect on the microbial community, the decreasing of treatment percentage of the wastewater treatment plant, as measured by COD removal, was expected. The COD in the influent was 70 mg L^−1^. The SBR without TiO_2_ addition can eliminate the pollutant efficiently with the residual COD as of 5 mg/L in the effluent. From table 2, the SBR with 0.05, 1 and 10 mg L^−1^ TiO_2_ provided an effluent with COD concentrations of 13, 15 and 23 mg L^−1^ after 30 days treatment duration, respectively. There is no statistical difference in the COD removal efficiency of the SBR with a TiO_2_ concentration less than 1 mg L^−1^. However, the impact of TiO_2_ loading is clearly seen when TiO_2_ concentration was greater than 10 mg L^−1^. The lowest percentage of COD removal (48.9%) was observed when the TiO_2_ concentration was the highest (50 mg L^−1^). These results indicated that the high concentration of TiO_2_ in the influent may cause the failure of pollutant removal in wastewater treatment plants.

**Table 2.** COD and TiO_2_ removal using activated sludge.

	COD in effluent		TiO_2_ in effluent	
Concentration of TiO_2_ (mg L^−1^)	(mg L^−1^)	% removal	(mg L^−1^)	% removal
Control	5	92.9	0.00	0.0
0.05	13.2	81.1	0.00	100.0
1	15.1	78.4	0.05	95.0
10	23.3	66.7	0.70	93.0
30	30.5	56.4	2.85	90.5
50	35.8	48.9	4.50	91.0

The impact of TiO_2_ nanoparticles on the efficiency of nutrient removal was also investigated. The concentrations of NH_4_^+^-N ions in the effluent using SBRs with 0.05, 2, and 10 mg L^−1^ TiO_2_ were 11, 13, and 10.8 mg L^−1^, which are approximately at the same level of NH_4_^+^-N (11.5 mg L^−1^) from the control reactor. Thus, our results suggest a negligible effect of TiO_2_ on nitrification in the SBR. The concentration of NO_2_-N and NO_3_-N in effluent from the SBR with 10 mg L^−1^ TiO_2_ was also comparable with the control reactor. In the control experiment, the effluent NO_2_ -N and NO_3_ -N concentrations were about 1.2 mg L^−1^ and 3.0 mg L^−1^, respectively. With the 10 mg L^−1^ TiO_2_, the nitrite concentration in the effluent was 1.0 mg L^−1^, while the nitrate rose to 3.2 mg L^−1^. The phosphorus concentrations in the effluent using SBRs with 10 mg L^−1^ TiO_2_ and the control experiment were 0.78 and 0.81 mg L^−1^, respectively. These nitrogen and phosphorus concentrations are only slightly different from the control experiment with the application of 10 mg L^−1^ of TiO_2_ to the SBR. Although the nutrient depletion in the present work was not clearly seen, this effect has been observed with the application of a higher concentration of TiO_2_ to the wastewater treatment. Zheng *et al* [27] found that 50 mg L^−1^ TiO_2_ exerted the significant decrease of total nitrogen removal efficiency after long-term exposure (70 days), whereas biological phosphorus removal was unaffected. Li *et al* [37] also reported that a high concentration of 200 mg L^−1^ TiO_2_ could cause a decreasing of nutrient concentration in the biological wastewater treatment plant. At a high concentration of TiO_2_ nanoparticles, abundant adsorption sites on the surface of TiO_2_ nanoparticles were accessible for the nutrient and trace metals. The available dissolved nutrients for the microorganisms may decrease due to the adsorption of these chemicals on the solid surface of nanoparticles. Deficiencies in nitrogen, phosphorus, or trace elements can cause the negative effects on growth and reproduction of healthy cells. The decrease of ammonia-oxidizing bacteria (AOB) and nitrite-oxidizing bacteria (NOB) in the presence of 50 mg L^−1^ TiO_2_ was reported previously [27]. In addition, the decrease of many protozoa species was reported in this work. Thus, the nutrient depletion that occurred in the biological wastewater treatment can lead to the indirect toxicity to the microorganisms in the system.

Regarding the efficiency of TiO_2_ removal using the SBR, a considerable removal of TiO_2_ nanoparticles by the activated sludge in the SBR configuration was observed during 30 days wastewater treatment. With the initial concentration in the range of 0.05–50 mg L^−1^, more than 90% of the TiO_2_ was removed. However, the TiO_2_ removal percentage varied depending upon the initial concentrations of TiO_2_. For example, 95% of the 1 mg L^−1^ TiO_2_ nanoparticles were easily removed, while the removal percentage decreased to 90–91% with 30–50 mg L^−1^ TiO_2_. Our results are in a good agreement with Kiser *et al* [4] who showed that 96% of the TiO_2_ was removed from the wastewater treatment plant when the TiO_2_ concentration was below 2 mg L^−1^. This information suggested that the TiO_2_ could be trapped in the bioflocs and, consequently, only a small amount of TiO_2_ escapes with the effluent.

## 4. Conclusions

The effect of TiO_2_ nanoparticles on biological wastewater treatment in an SBR has been investigated. The change in the microbial community, the biofloc in term of mixed liquor suspended solids, and the sludge volume index was monitored. The treatability performance in terms of chemical oxygen demand and TiO_2_ removal of the wastewater treatment plant were measured and reported. Results suggested that TiO_2_ nanoparticles tentatively exerted an adverse effect on the microbial population, causing the reduction of MLSS and SVI in the system. This information was confirmed by the detection of TiO_2_ in the biofloc component by SEM and EDS. The intrusion of TiO_2_ nanoparticles was found both on the surface and inside of the bioflocs. The reduction of total viable bacteria measured by heterotrophic plate counts was significant with the application of 50 mg L^−1^ TiO_2_ after two days of wastewater treatment. The respiration inhibition rate was increased, and the viability of the microbial population was reduced with the high concentration of TiO_2_.

Numbers of all investigated species of protozoa were diminished in the presence of TiO_2_. The higher concentration of TiO_2_, the more adverse effect on the protozoan community was evident. The TiO_2_ nanoparticles were ingested by the ciliates and rotifer protozoa as demonstrated by optical microscopy. The decreasing number of protozoa in a presence of TiO_2_ nanoparticles during 20 days of treatment is clearly described. The increase of COD in the system suggests a reduction in the efficiency of the wastewater treatment plant. The performance percentage based on COD removal during treatment of wastewater was reduced with a high concentration of TiO_2_. For TiO_2_ removal by SBR, more than 90% of the TiO_2_ with the concentration in the range of 0.05–50 mg L^−1^ was removed. The TiO_2_ could be trapped in the bioflocs, and consequently, only a small amount of TiO_2_ escapes with the treated water.

## References

[C1] Yuan Z, Li J, Cui L, Xu B, Zhang H, Yu C P (2013). Chemosphere.

[C2] Kumar A, Pandey A K, Singh S S, Shankez R, Dhawan A (2011). Free Radical Biol. Med..

[C3] Dhas S P, Shiny P J, Khan S, Mukherjee A, Chandrasekaran N (2013). J. Basic. Microbiol..

[C4] Kiser M A, Westerhoff P, Benn T, Wang Y, Pérez-Rivera J, Hristovski K (2009). Environ. Sci. Technol..

[C5] Musee N, Thwala M, Nota N (2011). J. Environ. Monit..

[C6] Labille J, Feng J, Botta C, Borschneck D, Sammut M, Cabie M, Auffan M, Rose J, Bottero J Y (2006). Environ. Pollut..

[C7] Pang X, Liu L, Liu Z H, Zhao Q (2005). IEEE-EMBS 2005: 27th Annu. Int. Conf. of the Engineering in Medicine and Biology Society.

[C8] Adams L K, Lyon D Y, Alvarez P J (2006). Water Res..

[C9] Block S S, Seng V P, Goswami D W (1997). J. Sol. Energy. Eng..

[C10] Sunada K, Watanabe T, Hashimoto K (2003). J. Photochem. Photobiol. A.

[C11] Reeves J F, Davies S J, Dodd N J F, Jha A N (2008). Mutat. Res..

[C12] Bar-Ilan O, Louis K M, Yang S P, Pedersen J A, Hamers R J, Peterson R E, Heideman W (2012). Nanotoxicology.

[C13] Battin T J, Fvd Kammer, Weilhartner A, Ottofuelling S, Hofmann T (2009). Environ. Sci. Technol..

[C14] Sun T Y, Gottschalk F, Hungerbühler K, Nowack B (2014). Environ. Pollut..

[C15] Ananpattarachai J, Kajitvichyanukul P, Seraphin S (2009). J. Hazard. Mater..

[C16] Lomer M C E, Thompson R P H, Commisso J, Keen C L, Powell J J (2000). Analyst.

[C17] Weir A, Westerhoff P, Fabricius L, Hristovski K, von Goetz N (2012). Environ. Sci. Technol..

[C18] American Public Health Association (APHA) 1999 *Standard Methods for the Examination of Water and Wastewater* 20th edn (New York: APHA)

[C19] Dehority B A (1984). Appl. Environ. Microbiol..

[C20] Liu Y, Li J, Qiu X F, Burda C (2007). J. Photochem. Photobiol. A.

[C21] Liu G, Wang D, Wang J, Mendoza C (2011). Sci. Total. Environ..

[C22] Cai Y, Stromme M, Welch K (2014). J. Biomater. Nanobiotechnol..

[C23] Wang Y, Westerhoff P, Hristovski K D (2012). J. Hazard. Mater..

[C24] Tiede K, Boxall A B A, Wang X M, Gore D, Tiede D, Baxter M (2010). J. Anal. At. Spectrom..

[C25] Sun X, Sheng Z, Liu Y (2013). Sci. Total Environ..

[C26] Jackson C R, Roden E E, Churchill P F (2000). Mol. Biol. Today.

[C27] Zheng X, Chen Y, Wu R (2011). Environ. Sci. Technol..

[C28] Muyzer G, Smalla K (1998). Antonie van Leeuwenhoek Int. J. Gen. Mol. Microbiol..

[C29] Sondi I, Salopek-Sondi B (2004). J. Colloid Interface Sci..

[C30] Morones J R, Elechiguerra J L, Camacho A, Holt K, Kouri J B, Ramirez J T, Yacaman M J (2005). Nanotechnology.

[C31] Thill A, Zeyons O, Spalla O, Chauvat F, Rose J, Auffan M, Flank A M (2006). Environ. Sci. Technol..

[C32] Madigan M T, Martinko J M (2006). Brock Biology of Microorganisms.

[C33] Mortimer M, Kasemets K, Kahru A (2010). Toxicology.

[C34] Jacobs M E, DeSouza L V, Samaranayake H, Pearlman R E, Siu K W, Klobutcher L A (2006). Eukaryotic Cell.

[C35] Nilsson J R (1977). J. Protozool..

[C36] Clément P, Wurdak E, Harrison F W, Ruppert E E (1991). Rotifera. Microscopic Anatomy of Invertebrates.

[C37] Li D, Cui F, Zhao Z, Liu D, Xu Y, Li H, Yang X (2013). Biodegradation.

